# Livestock grazing boosts plant diversity in the Greater Serengeti–Mara Ecosystem

**DOI:** 10.1002/eap.70214

**Published:** 2026-03-22

**Authors:** Yustina Kiwango, Rob Venderbos, Yuhong Li, Han Olff, Michiel P. Veldhuis

**Affiliations:** ^1^ Conservation Ecology Group, Groningen Institute for Evolutionary Life Sciences University of Groningen Groningen The Netherlands; ^2^ Tanzania National Parks Arusha Tanzania; ^3^ Institute of Environmental Sciences Institute, University of Leiden Leiden The Netherlands

**Keywords:** biodiversity conservation, biotic homogenization, Maasai pastoralist, protected area, scaling

## Abstract

Intensifying land use is a global threat to biodiversity, and livestock grazing—occupying 26% of terrestrial land—is one of such threats. Designated protected areas are one of the key conservation strategies to halt biodiversity loss, but their effectiveness is debated, in part because of data shortage at relevant spatial scales (>1 ha). We investigate how livestock grazing affects plant diversity in the Greater Serengeti–Mara Ecosystem, Tanzania. We recorded plant diversity in sites with and without livestock across different scales: from 1‐m^2^ plots to nine sites spanning a 600–1000 mm year^−1^ rainfall gradient. We find livestock grazing strongly increased alpha diversity (71%), with forb species richness increasing the most. Beta diversity decreased in pastoral areas both within and between sites. The total number of plant species at the landscape level was almost the same in Maasai rangelands (*N* = 210) and Serengeti National Park (*N* = 212), with a distinct vegetation composition and 85 unique species in livestock‐grazed areas. Our results suggest that livestock grazing—reducing light competition and eliminating fire—facilitates coexistence at local scales, biotically homogenizes across the rainfall gradient, yet provides novel niches at the landscape scale. We conclude that livestock grazing in the Greater Serengeti–Mara Ecosystem—with a long history of pastoralism—increases plant diversity by creating a diverse and distinct plant community, so that a mosaic of livestock‐grazed and ungrazed areas yields the highest value for conservation. We recommend rethinking current conservation strategies that focus on expanding protected area cover and upgrading protected area status, and instead invest in facilitating local communities in their efforts to sustainably coexist with nature.

## INTRODUCTION

Intensifying human land use—including agricultural intensification—forms a global threat to biodiversity and associated ecosystem services vital to human society (Allan et al., 2015; Barnosky et al., 2011; Chaudhary & Kastner, 2016; De Baan et al., 2013; Harrison et al., 2014; Oehri et al., 2017; van der Plas, 2019). Preventing biodiversity losses has been a central goal of nature conservation efforts (Eken et al., 2004; Jenkins et al., 2013; Rodrigues & Brooks, 2007), with protected areas—where human impact is limited—being one of the key strategies (Bruner et al., 2001; Geldmann et al., 2014; Laurance et al., 2012). Currently over one‐sixth of the global terrestrial surface falls within protected areas (Geldmann et al., 2019), and there is strong support for increasing this to 30% by 2030 (Baillie & Zhang, 2018; Dinerstein et al., 2019).

Whether expanding protected area size will increase biodiversity is not so straightforward as it may sound (Gatiso et al., 2022; Geldmann et al., 2019; Joppa et al., 2016; Rada et al., 2019), adding to a long debate on separating nature conservation from human land use versus including nature within areas dominated by people (Bram Büscher & Fletcher, 2020; Oldekop et al., 2016; Phalan et al., 2011). If we want to ensure the limited resources available for conservation make a difference, we must increase our understanding on the effectiveness of protected areas to prevent species loss and ecosystem degradation (Ferraro & Pattanayak, 2006). Yet, biodiversity conservation is facing a critical shortage of data to test the effectiveness of protected areas and their conservation strategies (Ferraro & Pattanayak, 2006; Kindsvater et al., 2018; Sutherland et al., 2004).

The Greater Serengeti–Mara Ecosystem is a network of core protected areas and human–wildlife coexistence landscapes and has a long history of anthropogenic influences, including fire and livestock grazing (Blench & MacDonald, 2011; Sinclair et al., 2008). Land use intensity has increased recently and livestock numbers have seen considerable growth over the past decades (Ogutu et al., 2016; Veldhuis et al., 2019). Livestock grazing can severely change vegetation composition, including woody plant encroachment, increased dominance of annual species, and increased abundance of non‐native species (Buisson et al., 2019; Mureithi et al., 2014; Nyarobi et al., 2022; O'Connor et al., 2014; Shezi et al., 2021; Stevens et al., 2017; Strum et al., 2015; Yé et al., 2015, 2017). At the same time, human land use—such as pastoralism—can provide novel habitat (Gregory et al., 2010; Killion et al., 2021; Landis, 2017; Maddox, 2003; Tscharntke et al., 2012) and be required to maintain grassland biodiversity (Yuan et al., 2016). How pastoralists affect plant diversity varies and remains poorly understood in this ecosystem because plant diversity is the outcome of a complex interplay among grazing, fire, rainfall, and scale (Huaranca et al., 2022; Wu et al., 2023).

Scale dependence has proven a complicating factor in understanding the interaction between plant communities and grazing, as several mechanisms influencing plant diversity are scale dependent (Anderson et al., 2007; Olff & Ritchie, 1998). Five main scale‐dependent mechanisms influencing diversity have been proposed: niche diversity at a ≤1 m^2^ scale, habitat diversity at a 1–10^3^ m^2^ scale, mass effect at a 10–10^6^ m^2^ scale, ecological equivalency at a >106 m^2^, and climates at even larger scales >10^8^ m^2^ (Anderson et al., 2007; Venevsky & Veneskaia, 2003). Herbivores affect vegetation diversity at both small and large scales by a multitude of mechanisms that affect extinction and immigration, including reduced dominance of abundant species, selection for grazing tolerant species, soil disturbance, seed dispersal, changed availability and heterogeneity of soil nutrients, and the introduction of patchiness with distinct plant communities (Bardgett & Wardle, 2003; Glenn & Collins, 1992; Li et al., 2021; C. Liu et al., 2016; Olff & Ritchie, 1998; Tonn et al., 2019).

Additionally, the effect of livestock grazing on vegetation diversity is to a large degree dependent on the local primary production. When production is low, large herbivores can have a neutral or even negative effect on plant species richness, while under more productive conditions, large herbivores are predicted to have a positive effect (Olff & Ritchie, 1998). On a local scale, the relationship between biodiversity and productivity is hump‐shaped, with the highest diversity at intermediate productivity (Anderson et al., 2004; Chase & Leibold, 2002). On a regional scale, however, productivity has been found to have a positive linear correlation with biodiversity (Chase & Leibold, 2002). In semi‐arid conditions, water availability limits production, and the mean annual precipitation levels have a strong positive correlation with the net primary production (Olff et al., 2002; Olff & Ritchie, 1998; Zhu & Southworth, 2013).

Experimental studies on the effects of grazing—including livestock—on plant diversity generally only cover small‐scale effects, as herbivore exclosures are limited in size (<1 ha) due to logistical constraints (Jia et al., 2018; Koerner et al., 2018). Comparisons of plant diversity between livestock‐grazed and ungrazed sites rarely include environmental gradients within a single ecosystem. Yet, protected areas—and their management—operate at the landscape scale. Effects of livestock grazing are expected to change with primary productivity (Olff & Ritchie, 1998). The effectiveness of protected areas should therefore be assessed at relevant scales for biodiversity conservation and include environmental gradients. These scale mismatches between conservation science and practice provide an important gap in understanding.

Here, we investigate how livestock grazing affects plant diversity across scales in the Serengeti National Park (SNP) and adjacent rangelands inhabited by Maasai pastoralists. We expected (1) livestock grazing will increase species richness at small scales—through relaxing competition between plants and increasing colonization rates—but decrease species richness at large scales—through selection for grazing‐adapted species (Jia et al., 2018; Mureithi et al., 2014; Olff & Ritchie, 1998; Vuorio et al., 2014). (2) Such biotic homogenization by livestock grazing is expected to decrease beta diversity, especially at larger scales (Chaneton & Facelli, 1991). (3) We expect livestock grazing to shift vegetation composition to more non‐native, annual, forbs, and armed species (Mohanbabu et al., 2023).

## MATERIALS AND METHODS

### Study area

**FIGURE 1:**
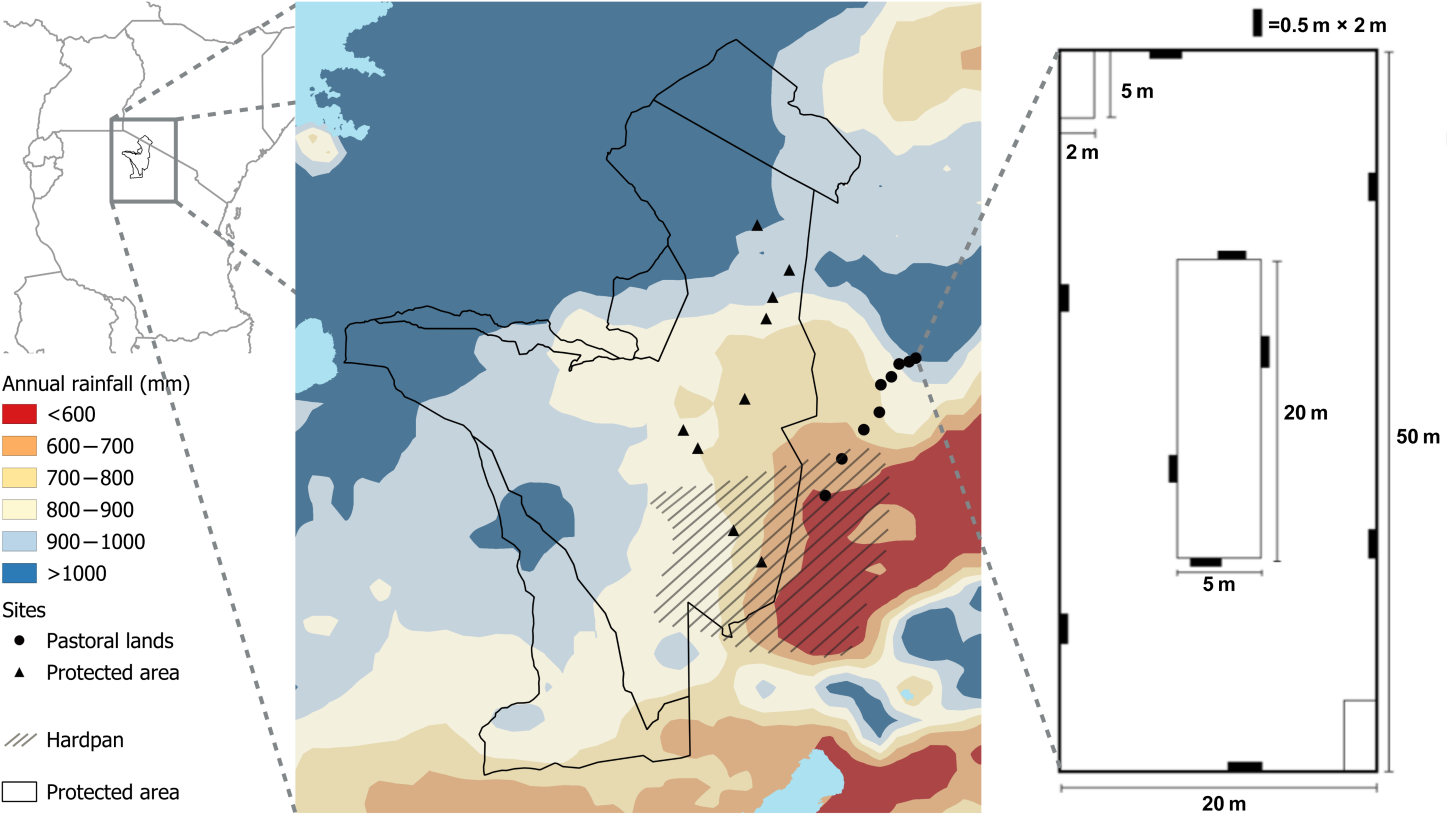
The Greater Serengeti–Mara Ecosystem. A large protected area network including the Serengeti National Park (SNP) and adjacent pastoral lands. Study sites were situated on the Tanzanian side of the ecosystem and covered both areas with livestock grazing (black dots) and without (black triangles) and covered a rainfall gradient (600–1000 mm year^−1^). Plant community composition was surveyed at each of the 18 sites using Modified‐Whittaker plots with 10 × 1 m^2^ subplots, 2 × 10 m^2^ subplots, and 1 × 100 m^2^ subplot, all nested within one 1000‐m^2^ plot. At low rainfall soil type transitions to volcanic ash—a shallow soil layer on a calcareous hardpan.

The Greater Serengeti–Mara Ecosystem exhibits a network of areas with different levels of protection status, including areas with a long history of human habitation and pastoralism (Figure 1) (Blench & MacDonald, 2011; Sinclair et al., 2008; Veldhuis et al., 2019). The SNP is a large core protected area in Tanzania where hunting, livestock grazing, and human settlement are prohibited. SNP is bordered by Loliondo Game Controlled Area (LGCA) and Ngorongoro Conservation Area (NCA) on the eastern side. LGCA and NCA are inhabited by Maasai pastoralists whose livestock graze the area (Bartels et al., 2017; Veldhuis et al., 2019). Cropping is prohibited in both areas, but does occur on a small scale in LGCA, as this rule is not enforced (Bartels et al., 2017). Grazing intensity within SNP varies between 0.2 (high rainfall) and 0.8 (low rainfall) (Veldhuis et al., 2019), where 0.8 indicates 80% of aboveground biomass is removed at the end of the growing season compared to sites with permanent exclosures. Grazing intensities in livestock‐grazed areas are higher and estimated to be >0.9 (personal observation).

SNP, LGCA, and NCA all support relatively high densities and diversity of large wild herbivores, although larger wild herbivores show higher densities in SNP, while smaller herbivores occur more in livestock‐grazed areas (de Jonge et al., 2022; Fryxell et al., 2022). Livestock densities outweigh wild herbivore densities, and consequently, the grazing intensity is much higher in LGCA. Fire has been eliminated for decades in most of LGCA and NCA (Hassan et al., 2008; Odadi et al., 2017; Veldhuis et al., 2019), while large parts of SNP burn every year (Probert et al., 2019). The decrease in fire frequency is caused by an increase in livestock numbers that reduces grass biomass to such an extent that burning is no longer possible (Probert et al., 2019; Veldhuis et al., 2019). Livestock in the area primarily consists of East African zebu cattle (*Bos indicus*), Red Maasai sheep (*Ovis aries*), East African goats (*Capra hircus*), and a small number of donkeys (*Equus africanus asinus*) used as pack animals (Bartels et al., 2017).

### Site selection and sampling

Eighteen sites were selected (Figure 1), of which half were grazed by livestock (LGCA and NCA) and half were only grazed by wildlife (SNP). Sites were spread out across a rainfall gradient to investigate the effects of productivity. All sites were positioned mid‐catena because the catena position—position along a sequence from hilltop to valley—strongly affects community assembly in this system (de Jonge et al., 2024), where water and nutrients accumulate downhill. Fourteen sites were positioned on granite‐derived soils—the most common soil type in the area. The four remaining sites—two with and two without livestock—were positioned in low rainfall areas, which coincides with a soil type transition to volcanic ash—a shallow soil layer on a calcareous hardpan. The shallow soils increase the drought effects, further reducing the moisture available to plants. Mean annual rainfall per site was estimated using Climate Hazards Group InfraRed Precipitation with Station data (CHIRPS) from 2000 until 2021 (de Jonge et al., 2022; Funk et al., 2015).

We surveyed vegetation on each site in the wet season of 2022 between March 14 and May 13. At each site, plant species richness was assessed at 1, 10, 100, and 1000 m^2^ scales using Modified‐Whittaker plots (Figure 1) (Anderson et al., 2007; Ghorbani et al., 2011; Stohlgren et al., 1995). The Modified‐Whittaker plots included ten 1‐m^2^ subplots, two 10‐m^2^ subplots, and one 100‐m^2^ subplot. These smaller subplots were all nested in the 1000‐m^2^ plot. In each subplot, all plant species (including seedlings) were identified to the lowest taxonomic classification possible. In the 1000‐m^2^ plot, species were recorded that were not encountered in the subplots, by systematic walking through the entire plot with three observers for 30 min. The nomenclature of “kew, plants of the world online” was used (POWO, 2022).

### Species characteristics

Species characteristics were extracted from “kew, plants of the world online”, favoring information from the Flora of Tropical East Africa (Milne‐Redhead et al., 2012; POWO, 2022). We selected functional group (forb, grass, sedge, succulent, and woody), predominant lifespan (annual or perennial), presence of mechanical defenses (spikes and thorns), and whether or not they are native in Tanzania as key properties to explain the effects of livestock grazing on plant diversity. When information was not available for a species, records of that species were not considered in the analysis. Information was available for the majority of species: functional group (97%), predominant lifespan (67%), mechanical defenses (75), and nativeness (72%).

### Analysis

We analyzed data in R4.4.0 (R Core Team, 2024), using *tidyverse* for data management (Wickham et al., 2019). First, we used species–area relationships (SAR) to investigate trends in species richness. For each site, we regressed the observed number of species against the log‐transformed area of observation (1–1000 m^2^). The intercept of the regression line represents the predicted number of species per unit area—alpha diversity—and the slope indicates how quickly species richness increases as area increases. We investigated the intercept and slope of the SAR change with livestock presence using two‐way ANOVAs. Soil type was included as covariate in the model. We also tested whether rainfall affected the intercept and slope of the SARs, using linear regression models with rainfall and livestock presence as predictors. The distribution of the data and assumptions of the models were inspected visually.

Second, beta diversity was assessed by producing non‐metric multidimensional scaling (NMDS) using *vegan* for the ten 1‐m^2^ subplots per site (Oksanen et al., 2022). Additionally, the average Jaccard dissimilarity index per site was determined using the *vegdist* function from the *vegan* package using the 1‐m^2^ subplots. Community dissimilarity has a high correlation with beta diversity and can be used to compare treatments (Chase & Leibold, 2002). We tested whether between‐site beta diversity—Jaccard dissimilarity of 1000‐m^2^ plots—varied with livestock presence using ANOVAs with soil type as covariate. Furthermore, we tested whether beta diversity varied with rainfall using a linear regression model with rainfall and livestock presence as predictors. To determine whether differences in plant species composition between sites increased with rainfall differences, we subtracted annual rainfall between sites (rainfall differences) and regressed this against the Jaccard dissimilarity between sites.

Third, we tested whether livestock grazing affected the number and proportion of different functional groups of plant species in the community per site, using linear regression models with livestock presence and rainfall as explanatory variables, and ANOVAs with livestock presence and soil type as explanatory variables in each model.

## RESULTS

**FIGURE 2:**
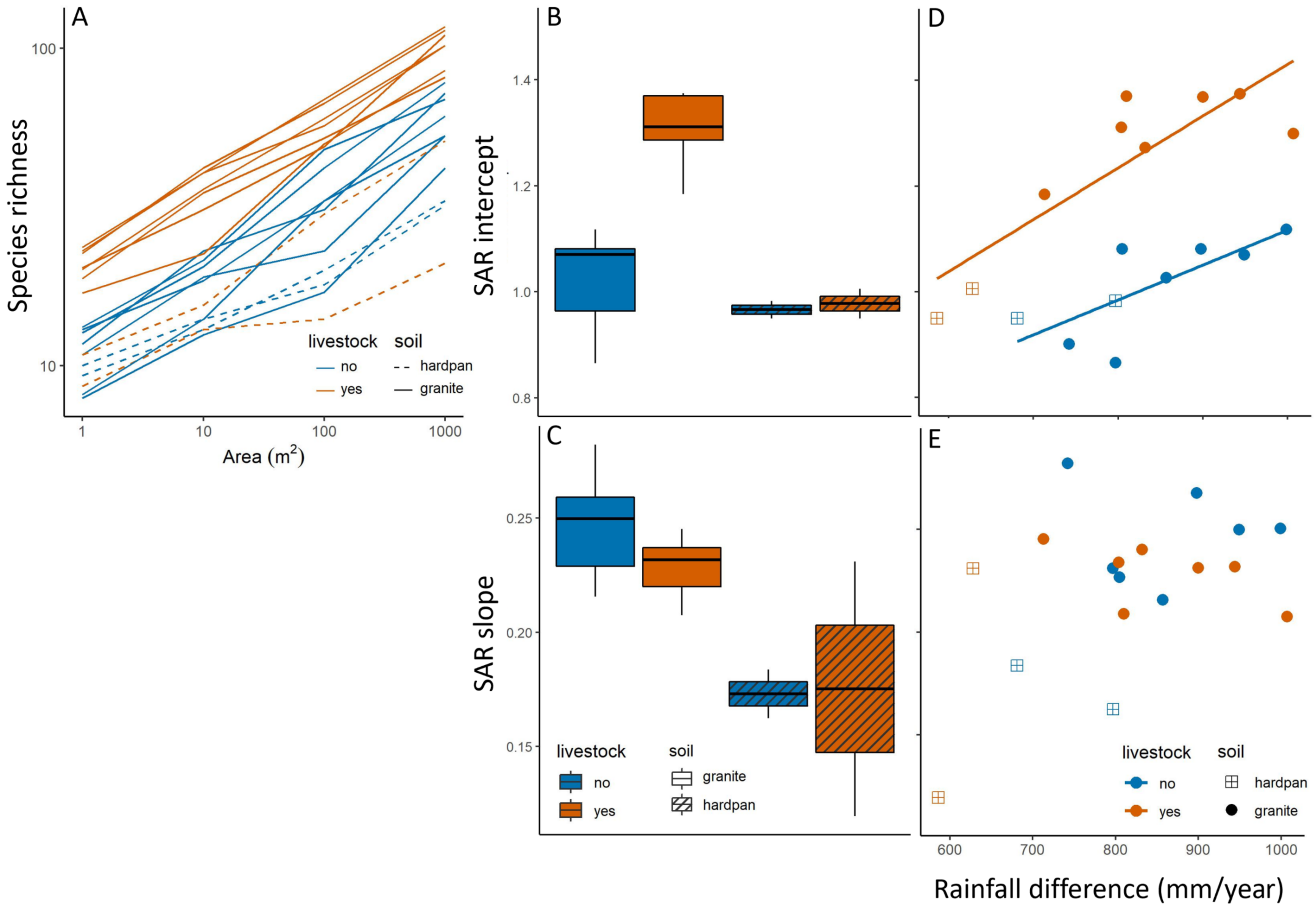
The effect of livestock grazing, rainfall, and soil type on vegetation species–area relationships (SAR) for 18 sites in the Greater Serengeti–Mara Ecosystem. (A) SAR for each of the 18 sites surveyed using Modified‐Whittaker plots (see Figure 2). Half of the sites were intensively grazed by livestock (red), while the other half was only accessible for wild herbivores (blue). Fourteen sites are located on granite‐derived soils (solid lines), while four sites were positioned on volcanic soils with a shallow calcareous hardpan (dashed lines). (B) Livestock‐grazed sites had significantly higher SAR intercepts (ANOVA: *F*_1,14_ = 37.6, *p* < 0.001), but only on granite soils (*F*_1,14_ = 18.6, *p* < 0.001) resulting in a significant interaction between livestock grazing and soil type (*F*_1,14_ = 9.70, *p* = 0.007). (C) The SAR slope was not different for areas with or without livestock (ANOVA: *F*_1,15_ = 1.13, *p* = 0.30), but slopes were steeper on granite soils (*F*_1,15_ = 16.3, *p* = 0.001). (D) SAR intercepts increased with rainfall (LM: *t* = 5.6, *p* < 0.001) and were higher for livestock‐grazed areas (*t* = 7.19, *p* < 0.001). (E) SAR slopes did not change with rainfall (LM: *t* = 1.75, *p* = 0.10) or livestock grazing (*t* = −0.55, *p* = 0.59).

On average, livestock grazing increased species richness from 10.7 to 18.3 in 1‐m^2^ plots (Figure 2A). This positive effect of livestock on alpha diversity—indicated by the intercept of the SAR—was only found on granite soils, whereas sites with volcanic soils and shallow calcareous hardpan showed no differences in alpha diversity with livestock grazing (Figure 2B). Livestock did not affect the slope of the SAR, so that the increased species richness was consistent across scales ranging from 1 to 1000 m^2^ (Figure 2C). Species richness increased faster with scale on granite sites than sites with a hardpan, indicated by a higher SAR slope (Figure 2C). Alpha diversity increased with rainfall (Figure 2D), and this effect was similar for areas with and without livestock, indicated by a nonsignificant interaction term between livestock presence and rainfall (LM: *t* = 0.98, *p* = 0.34). Rainfall did not affect the SAR slope, so species richness increased at similar rates across scales independent of rainfall (Figure 2E). These effects of rainfall were robust to exclude the hardpan sites.

**FIGURE 3:**
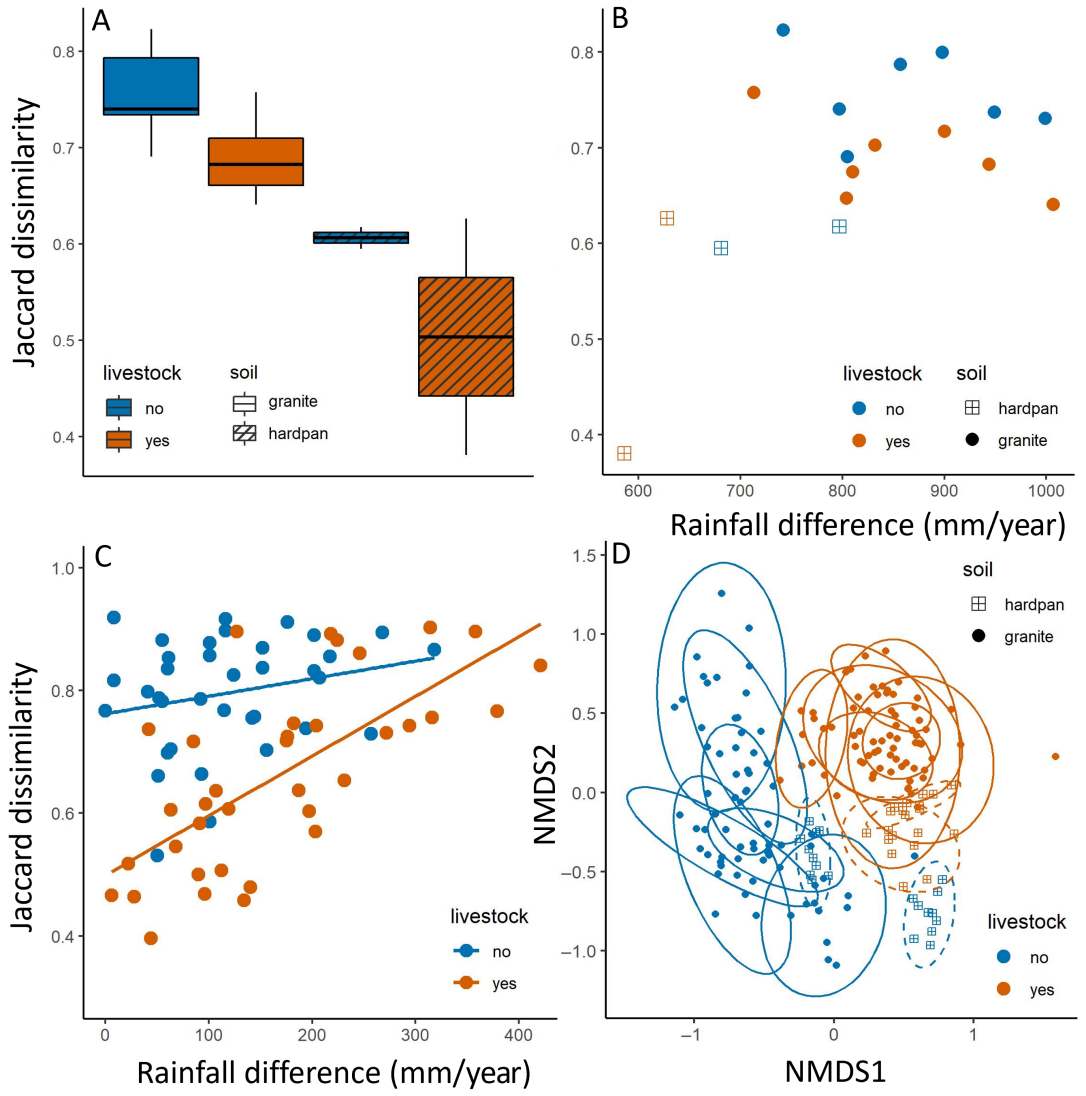
The effect of livestock grazing, rainfall, and soil type on within (A, B) and between‐site (C, D) beta diversity in the Greater Serengeti–Mara Ecosystem. (A) Livestock‐grazed sites had lower beta diversity when comparing ten 1‐m^2^ plots at each site (ANOVA: *F*_1,14_ = 7.12, *p* = 0.02), and granite soils showed higher beta diversity than hardpan soils (*F*_1,14_ = 23.2, *p* < 0.001). There was no significant interaction between livestock grazing and soil type (*F*_1,14_ = 0.22, *p* = 0.65). (B) Annual rainfall did not affect within‐site beta diversity (LM: *t* = −0.50, *p* = 0.62), and the effect of rainfall did not interact with livestock grazing (*t* = 0.35, *p* = 0.73) or soil type (*t* = 1.47, *p* = 0.17). (C) Species turnover between sites—indicated by Jaccard dissimilarity index—was higher for areas without livestock grazing (LM: *t* = −5.97, *p* < 0.001). For areas with livestock grazing, between‐site beta diversity increased steadily with a difference in rainfall (LM: *t* = 5.67, *p* < 0.001), while in areas without livestock, there was no effect of rainfall (*t* = 1.42, *p* = 0.16), resulting in a significant interaction between livestock and rainfall in the full model (LM: *t* = 2.55, *p* = 0.01). (D) An ordination—using non‐metric multidimensional scaling (NMDS)—shows that livestock‐grazed sites cluster together and show a large overlap compared to areas without livestock. Each dot represents a 1‐m^2^ plot and ellipses represent sites.

Within‐site beta diversity—expressed as Jaccard dissimilarity of the ten 1‐m^2^ plots within each site—was higher on granite soils than hardpan soils (Figure 3A). Livestock decreased the variation in species composition between plots (Figure 3A). Annual rainfall did not affect within‐site beta diversity (Figure 3B), and this result was consistent when only selecting granite sites (LM: *t* = −1.53, *p* = 0.15). Between‐site beta diversity—expressed as Jaccard dissimilarity of 1000‐m^2^ plots between sites—was also higher for sites without livestock grazing (Figure 3D), where species turnover rates were very high irrespective of rainfall differences between sites. At sites with livestock, plant communities were relatively similar to sites with similar annual rainfall, and plant communities differed more when sites were further spaced out across the rainfall gradient (Figure 3C). The clustering of livestock‐grazed sites in an ordination of plant communities (Figure 3D) shows the increased similarity of plant communities. Sites without livestock show less overlap and are spread out further.

In total, 210 species were recorded in sites with livestock and 212 at sites without livestock grazing. One hundred and twenty‐eight species were found under both management types, so each area exhibited no less than 82 unique species, bringing the total of plant species to 294.

**FIGURE 4:**
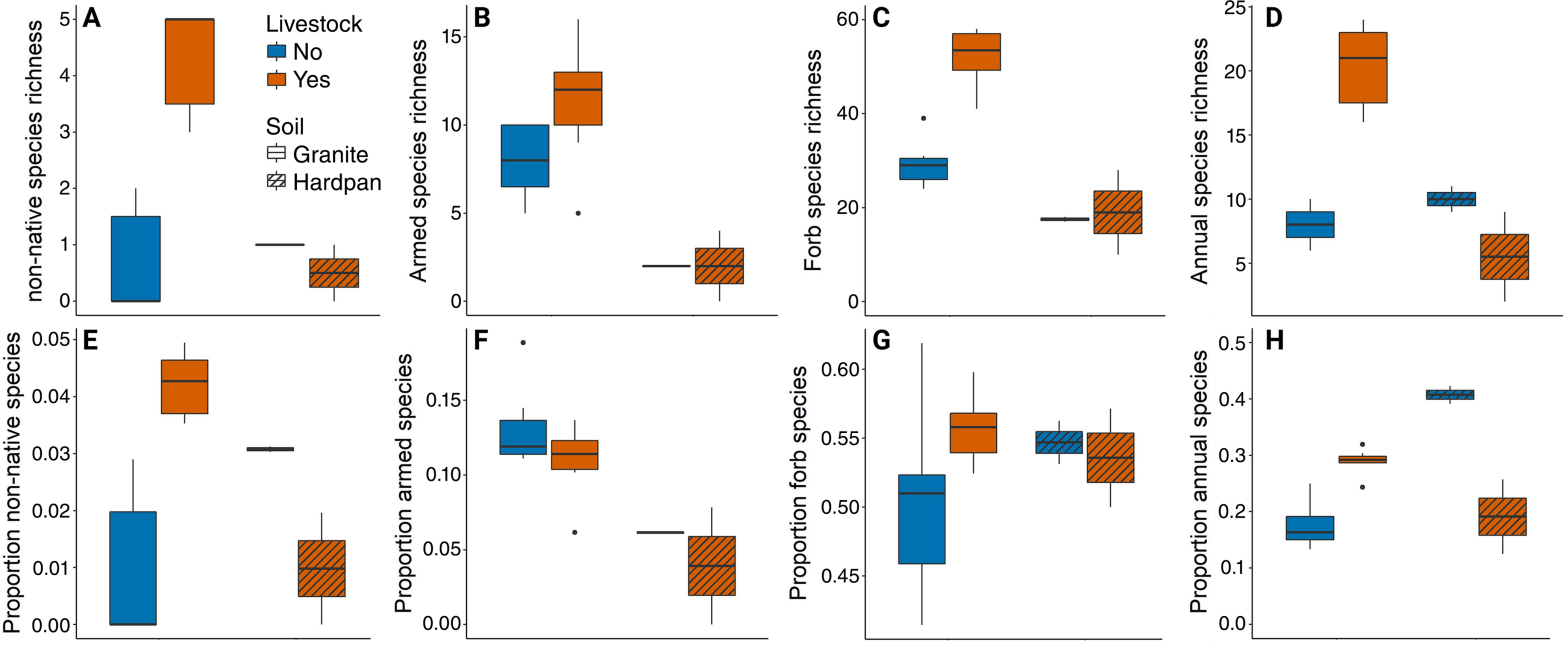
Livestock grazing effects on the number (A–D) and proportion (E–H) of different functional groups of plant species. (A) Livestock grazing increased non‐native species richness (ANOVA, *F*_1,14_ = 39.4, *p* < 0.001), but only on granite soils, yielding a significant interaction term (*F*_1,14_ = 15.9, *p* = 0.001). (B) Plants with mechanical defenses were not affected by livestock presence (ANOVA, *F*_1,15_ = 3.81, *p* = 0.07), and were present more on granite‐derived soils (*F*_1,15_ = 23.5, *p* < 0.001). (C) Forb species richness increased with livestock grazing on granite‐derived soils (ANOVA, *F*_1,14_ = 38.3, *p* < 0.001), but not on hardpan soils yielding a significant interaction term (*F*_1,14_ = 9.29, *p* = 0.008). (D) Livestock grazing increased the proportion of annual species on granite‐derived soils (ANOVA, *F*_1,14_ = 44.4, *p* < 0.001), but not on hardpan soils yielding a significant interaction term (*F*_1,14_ = 29.5, *p* < 0.001). (E) Proportion of non‐native species increased with livestock grazing on granite‐derived soils (ANOVA: *F*_1,12_ = 35.8, *p* < 0.0001) but not on hardpan soils, yielding a significant interaction in a model containing both livestock and soils (*F*_1,14_ = 21.8, *p* < 0.001). (F) Proportion of plants with mechanical defenses was not affected by livestock presence (ANOVA, *F*_1,15_ = 2.91, *p* = 0.11), and was higher on granite‐derived soils (*F*_1,15_ = 20.4, *p* < 0.001). (G) Proportion of forb species was not affected by livestock grazing (ANOVA, *F*_1,15_ = 3.07, *p* = 0.10) or soil type (*F*_1,15_ = 0.20, *p* = 0.66). (H) Proportion of annual plant species increased with livestock grazing on granite‐derived soils (ANOVA, *F*_1,14_ = 4.64, *p* = 0.05), but seemed to decrease on hardpan soils, yielding a highly significant interaction term (*F*_1,14_ = 54.1, *p* < 0.001).

Livestock grazing effects on vegetation composition were also more pronounced on granite‐derived soils (Figure 4; Appendix S1: Figure S1), although this is partly confounded by the lower sample size of sites on hardpan soils (*n* = 4 vs. *n* = 14). Eight non‐native species were identified, of which seven were recorded in sites with livestock and five in sites without livestock. Livestock presence increased both the number and the proportion of non‐native plants on sites with granite‐derived soil (Figure 1A,E). Overall, the number of non‐native species recorded was low and could not explain the increased plant diversity in livestock‐grazed sites.

The proportion and number of armed—that is, spines and thorns—plant species were higher on granite‐derived soils but not affected by livestock grazing (Figure 4B,F). Armed species richness generally increased with rainfall (LM: *t* = 2.87, *p* = 0.01), but the proportion of armed plants did not (LM: *t* = 1.74, *p* = 0.10). The number and proportion of annual species increased with livestock grazing on granite‐derived soils but not on hardpan soils (Figure 4D,H). Annual species richness increased with rainfall in areas with livestock (LM: *t* = 2.88, *p* = 0.02), while the proportion of annual species remained constant (LM: *t* = 1.91, *p* = 0.10). In areas without livestock, annual species richness did not change with rainfall (LM: *t* = −0.17, *p* = 0.87), so that the proportion of annual species decreased towards higher rainfall sites in areas without livestock (LM: *t* = −2.10, *p* = 0.07).

Forbs contributed most to the increased plant richness in livestock‐grazed areas—53.3 versus 29.3 species on average on granitic soils (Figure 4C)—but also grass and woody species richness increased while sedge species numbers remained stable (Appendix S1: Figure S1). The proportion of species of all four functional groups did not change with livestock grazing (Figure 4G; Appendix S1: Figure S1E–H).

## DISCUSSION

We surveyed plant diversity in the SNP and adjacent pastoral rangelands to assess how livestock changes biodiversity patterns. We aimed to better understand scale‐dependent changes across a rainfall gradient. We found that livestock grazing severely increased (>70%) plant diversity at the plot and site levels, but this effect disappeared at larger scales because sites were more similar in species composition in pastoral rangelands. Livestock grazing led to a distinct vegetation composition—rich in forbs and annuals—so that a combination of protected area and pastoral rangeland optimizes plant diversity at the landscape scale.

The effect of livestock grazing on plant diversity varies (Huaranca et al., 2022; Wu et al., 2023), but strong increases in plant diversity are commonly found (Fensham et al., 1999; Heady, 1966; Sala et al., 1986; Yuan et al., 2016). The typical increase of forbs and annuals also corroborates previous findings in other regions of the world (Dorrough et al., 2004; Pettit et al., 1995; Stahlheber & D'Antonio, 2013; Yates et al., 2000). Grazing favors shorter‐statured plant species that allocate a large portion of their resources below‐ground (Belsky, 1992; Bullock et al., 2001; Díaz et al., 2007; Evju et al., 2009; Milchunas & Lauenroth, 1993), suggesting livestock grazing reduced competition between plant species for light, converting a tall perennial species community into a community dominated by short annuals and forbs (Belsky, 1992).

This could also explain why livestock grazing had limited impact on diversity in hardpan soils, where vegetation biomass is low due to limited soil water availability—a combination of low rainfall and shallow soils (de Wit, 1978; McNaughton, 1985). Plant diversity increased with rainfall—a strong proxy for productivity in this system (McNaughton, 1985)—both in the national park and pastoral lands, which aligns with general patterns between productivity and biodiversity (Mittelbach et al., 2001). In contrast to our expectations, plant diversity did not decrease at high rainfall sites—yielding the typical hump‐shaped pattern—but at best leveled off. Possibly, our productivity gradient should be further extended before this pattern can be observed. Alternatively, the high top‐down pressure of fire inside protected areas and livestock in the pastoral lands prevents competitive exclusion at high productivity in both areas. Within‐site beta diversity did not change with rainfall, supporting this latter hypothesis of lack of competitive exclusion.

Livestock grazing decreased beta diversity—within and between sites—suggesting homogenization overrides patch formation. Both grazing by wild herbivores (McNaughton, 1985) and fire characteristics—size, radiative power, frequencies (Probert et al., 2019)—align with environmental gradients such as rainfall and possibly even increase habitat diversity along those gradients through ecological feedback loops (Hempson et al., 2019; Veldhuis et al., 2018). Livestock grazing has the opposite effect, providing a strong filter for the plant composition in this system that overrides environmental gradients and reduces stochastic relative to deterministic community assembly processes (Chase, 2010). The scaling of livestock effects on plant diversity with positive alpha diversity and negative beta diversity has been previously observed (Chaneton & Facelli, 1991; Olff & Ritchie, 1998). Biodiversity effects frequently switch directions across scales (Chase et al., 2018), highlighting the importance of a multiscale approach to achieve a deeper understanding of the effectiveness of conservation strategies.

The total plant diversity in protected areas and pastoral lands was the same, but the botanical composition differed strongly, with each area harboring >80 unique species. Heterogeneity is an important feature of the Greater Serengeti–Mara Ecosystem, with migrating mammal populations making optimal use of the resources available in the distinct parts of the system (Avgar et al., 2014; Hopcraft et al., 2014). Many ungulate species utilize and some—Grant's gazelle, giraffe—even prefer pastoral rangelands over protected areas, at least for part of the year (Bhola et al., 2012; de Jonge et al., 2022). Other taxonomic groups—including grasshoppers, birds—have been shown to benefit from the traditional pastoralist practices of the Maasai, which can create a matrix of varying land use intensity (Gregory et al., 2010; Uyehara et al., 2016; Vuorio et al., 2014). These effects are site dependent (Filazzola et al., 2020; Huaranca et al., 2022; Jia et al., 2018; Wu et al., 2023) and often peak at intermediate grazing (Yuan et al., 2016).

Our study supports evidence‐based decision‐making in the management of the Greater Serengeti–Mara Ecosystem and questions the global push for increased protected area size. We show that a mosaic of areas with variation in management—such as livestock grazing—boosts plant diversity by providing novel habitat in the Greater Serengeti–Mara Ecosystem, contributing to the overall heterogeneity of the region at large. The effectiveness of conservation strategies might thus increase if we move away from focusing on expanding protected area cover and instead invest in facilitating local communities in their efforts to sustainably coexist with wildlife.

## AUTHOR CONTRIBUTIONS

Yustina Kiwango, Han Olff, and Michiel P. Veldhuis designed research; Rob Venderbos and Yuhong Li collected field data; Yustina Kiwango., Rob Venderbos, and Michiel P. Veldhuis analyzed data; and Yustina Kiwango, Rob Venderbos, and Michiel P. Veldhuis wrote the paper.

## CONFLICT OF INTEREST STATEMENT

The authors declare no conflicts of interest.

## Supporting information


Appendix S1.

## Data Availability

Data and code (Veldhuis, 2025) are available in Zenodo at https://doi.org/10.5281/zenodo.15012005.
