# Supplementary material for: Livestock grazing boosts plant diversity in the Greater Serengeti–Mara Ecosystem
Source: Ecol Appl. 2026 Mar 22;36(2):e70214. doi: 10.1002/eap.70214 (PMC13005998; doi:10.1002/eap.70214)
Supplement: Supplementary file 1 — Appendix S1. [file EAP-36-e70214-s001.pdf]

Appendix S1

Livestock grazing boosts plant diversity in the Greater Serengeti-Mara Ecosystem

Yustina Kiwango, Rob Venderbos, Yuhong Li, Han Olff and Michiel P. Veldhuis

Ecological Applications

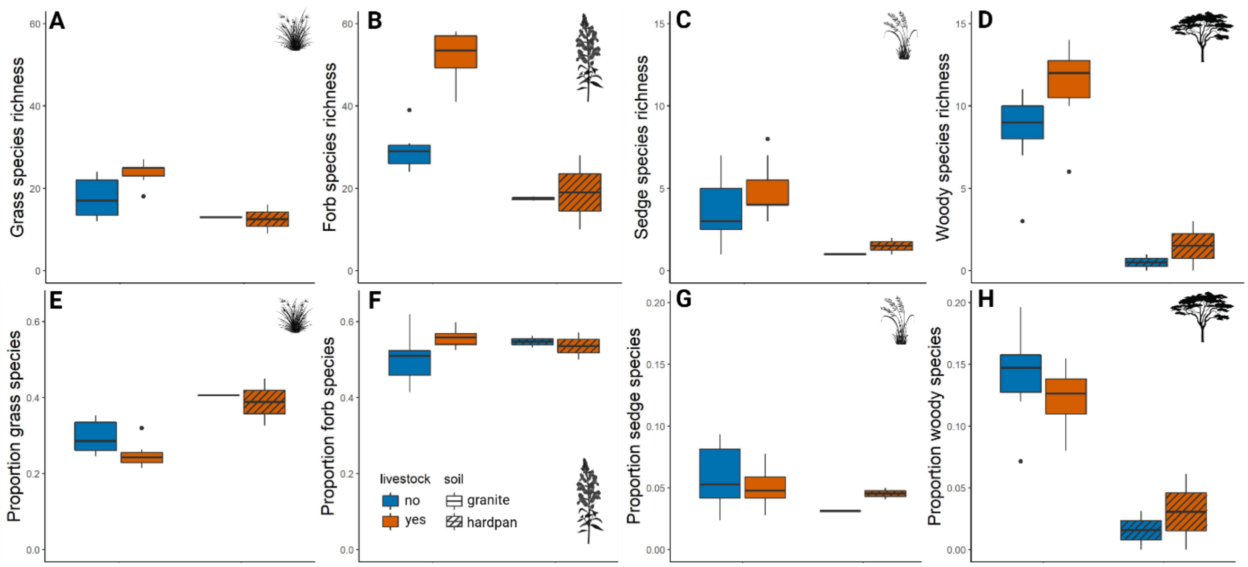

Figure S1. Livestock grazing effects on the number and proportion of different functional groups of plant species. **A**) Grass species richness increased with livestock grazing (ANOVA, $F_{1,15}$ = 5.34, P = 0.03), and was higher on granite soils ($F_{1,15}$ = 11.3, P = 0.004). **B**) Forb species richness increased with livestock grazing on granite-derived soils (ANOVA, $F_{1,14}$ = 38.3, P < 0.001), but not on hardpan soils yielding a significant interaction term ($F_{1,14}$ = 9.29, P = 0.008). **C**) Sedge species richness was higher on granite-derived soils (ANOVA, $F_{1,15}$ = 8.76, P = 0.009), but not affected by livestock grazing ($F_{1,15}$ = 1.37, P = 0.26). **D**) Woody species richness increased with livestock grazing (ANOVA, $F_{1,15}$ = 4.93, P = 0.04) and was higher on granite-derived soils ($F_{1,15}$ = 34.2, P < 0.001). **E**) Proportion of grass species was lower on granite-derived soils (ANOVA: $F_{1,15}$ = 26.5, P < 0.001) but not affected by livestock grazing ($F_{1,15}$ = 4.10, P = 0.06). **F**) Proportion of forb species was not affected by livestock grazing (ANOVA, $F_{1,15}$ = 3.07, P = 0.10), or soil type ($F_{1,15}$ = 0.20, P = 0.66). **G**) Proportion of sedge species was not affected by livestock grazing (ANOVA: $F_{1,15}$ = 0.15, P = 0.70) or soil type ($F_{1,15}$ = 2.10, P = 0.17). **H**) Proportion of woody species was not affected by livestock grazing (ANOVA, $F_{1,15}$ = 0.49, P = 0.50), but higher on granite-derived soils ($F_{1,15}$ = 34.0, P < 0.001). Figure created by M.P. Veldhuis (2026) using icons of plant illustrations from BioRender (https://BioRender.com/s2bsl7z).
